# Mapping HIV prevalence using population and antenatal sentinel-based HIV surveys: a multi-stage approach

**DOI:** 10.1186/s12963-015-0055-z

**Published:** 2015-09-02

**Authors:** Samuel Manda, Lieketseng Masenyetse, Bo Cai, Renate Meyer

**Affiliations:** Biostatistics Unit, South African Medical Research Council, Pretoria, South Africa; School of Mathematics, Statistics & Computer Science, University of KwaZulu-Natal, Pietermaritzburg, South Africa; Department of Epidemiology and Biostatistics, University of South Carolina, Columbia, USA; Department of Statistics, University of Auckland, Auckland, New Zealand

## Abstract

**Background:**

Sound public health policy on HIV/AIDS depends on accurate prevalence and incidence statistics for the epidemic at both local and national levels. However, HIV statistics derived from epidemiological extrapolation models and data sources have a number of limitations that may lead to under- or overestimation of the epidemic. Thus, adjustment techniques need to be employed to correctly estimate the size of the HIV burden.

**Methods:**

A multi-stage methodological approach is proposed to obtain HIV statistics at subnational levels by combining nationally population-based and antenatal clinic HIV data.

The stages range from computing inverse probability weighting (IPW) for consenting to HIV testing, to HIV status prediction modelling, to the recently developed Bayesian multivariate spatial models to jointly model and map multiple HIV risks.

The 2010 Malawi Demographic and Health Survey (MDHS 2010) and the 2010 Malawi Antenatal Clinic (ANC 2010) Sentinel HIV data were used for analyses. Gender, residence, employment, marital status, ethnicity, condom use, and multiple sex partners were considered when estimating HIV prevalence.

**Results:**

The observed MDHS 2010 HIV prevalence among people aged 15–49 years was 10.15 %, with 95 % confidence interval (CI) of (9.66, 10.67 %). The ANC 2010 site HIV prevalence had a median of 10.63 %, with 95 % CI ranging from 1.85–24.09 %. The MDHS 2010 prevalence was 10.61 % (9.9, 11.33 %) and 10.19 % (9.69, 10.71 %) using the HIV weight and IPW, respectively. After predicting the HIV status for the non-tested subjects, the overall MDHS 2010 HIV prevalence was 11.05 % (10.80, 11.30 %). Higher HIV prevalence rates were observed in the mostly Southern districts, where poverty and population density levels are also comparatively high. The excess risk attributable to ANC HIV was much larger in the central-eastern and northern parts of the country.

**Conclusions:**

Inverse Probability Weighting combined with an appropriate HIV prediction model can be a useful tool to correct for non-response to HIV testing, especially if the number of tested individuals is very minimal at subnational levels. In populations where most know their HIV status, population-based HIV prevalence estimates can be heavily biased. High-coverage antenatal clinics’ surveillance HIV data would then be the only important HIV data information sources.

## Introduction

Robust design of public health policy responses to the HIV epidemic depends on accurate estimates of HIV prevalence at both local and national levels [1, 2]. Precise estimates of HIV prevalence are important for a better understanding of correlations between HIV status and geographical characteristics, effective targeting of public health interventions and resource allocation, as well as assessment of progress made in controlling the epidemic [3]. According to Boerma et al. [4], national population-based surveys for HIV provide more accurate information on HIV prevalence than other data sources. However, these national surveys still remain scarce in Africa as they are costly to implement [5].

Therefore, most countries in Africa, especially those with a generalized epidemic, rely on ANC sentinel surveillance systems to monitor trends and progress made against the HIV epidemic [1–3]. ANC surveillance has well-known and documented limitations and biases that may lead to under- or overestimation of HIV prevalence [1–3]. These limitations include an excess of sentinel ANC sites in urban areas and other readily accessible locations; the exclusion of men; and a potential difference in HIV prevalence between pregnant and non-pregnant women, among others. Moreover, the ANC surveys represent only pregnant women, who by definition have been sexually active and are of reproductive age, and women with potentially HIV-associated infertility are not captured. Thus, a number of countries have started conducting nationally representative population-based surveys that include HIV testing. For example, Demographic and Health Surveys that include HIV testing have been conducted in over 30 countries in Africa, Asia, Latin America, and Eastern Europe [1, 3].

However, even nationally representative population surveys suffer from possibly significant non-response [5]. For instance, in the 2004 Malawi Demographic Health Survey, 30 % of women and 37 % of men in the HIV subsample refused to consent to HIV testing (NSO, Malawi and ORC Macro, 2005) [6]. Nonparticipation in an HIV test due to absenteeism or refusal to be tested creates several associated biases. According to the 2012 nationally representative household HIV survey in South Africa, only 67.5 % (28,997) of eligible individuals consented to HIV testing, with Black Africans, at 73.3 %, having the highest HIV testing response among the four race groups [7]. One of the main reasons for a subject to refuse HIV testing is their HIV status knowledge [1]. In order to account for the effects of non-response, HIV prevalence estimates could potentially be corrected using statistical procedures as we describe below. Nonetheless, nationally representative surveys are a marked improvement as they provide superior estimates of HIV prevalence if accompanied by high HIV testing uptake [1].

On the other hand, integrated demographic and epidemiological extrapolation and statistical models are used to measure the HIV/AIDS epidemic. These have evolved and become more sophisticated with increased quality and abundance of underlying data sources [3]. Most of these techniques use the available data, including ANC HIV, mortality, and fertility data to estimate the size of the HIV burden at both national and subnational levels. Most national projections of HIV prevalence and incidence rates in sub-Saharan Africa are derived from the Spectrum/Estimation and Projection Package (EPP) software [8]. Some of the models are local, such as the Actuarial Society of South Africa (ASSA) AIDS and Demographic and the THEMBISA models [9]. These models have their own limitations due to varying input variables such as population estimates, HIV prevalence rates, number of patients on antiretroviral drugs (ART), fertility and mortality rates, and assumptions of underlying statistical models on mortality.

This paper proposes a multi-stage approach to obtain optimal HIV statistics using population-based HIV surveys. First, inverse probability weights (IPWs) are derived from subject-level probabilities of accepting HIV testing based on an appropriate HIV test response regression model. Second, using the observed HIV status of the HIV-tested sample, an HIV prediction model is determined. Third, the HIV prediction equation is used to impute HIV status for subjects who refused HIV testing and survey interviews. Using various subject-level HIV weights (IPW and survey response weights), weighted subnational estimates of HIV prevalence are computed using all the subjects, some with observed and some with imputed HIV status. Finally, recently developed Bayesian multivariate spatial models are used to obtain smoothed HIV prevalence maps at the appropriate subnational level by combining population-based and ANC HIV data sources.

As an application, we used the 2010 Malawi Demographic and Health Survey (MDHS 2010) and the 2010 Malawi ANC HIV data in the multivariate spatial analyses. As in most sub-Saharan African countries, Malawi has been monitoring HIV prevalence predominantly through antenatal clinic (ANC) sentinel surveillance [10]. We adopted the conditional predictive ordinate (CPO), which is the marginal posterior predictive density, for model validation and accuracy. In particular, by accounting adequately for non-response to HIV testing, we hope that the nationally representative survey used in this study that includes HIV testing will provide more accurate information on HIV prevalence than sentinel ANC-based survey data, as advocated by Boerma et al. [4].

Malawi is a country in sub-Saharan Africa (SSA), a region heavily affected by the epidemic compared to other regions anywhere else, with an estimated 22.4 million people living with HIV [11]. Despite recent declines in HIV prevalence, Malawi remains among the group of countries with the largest HIV epidemics in the world. The country’s HIV prevalence in adults (15–49 years) peaked at 15 % in 1998 and began stabilizing, reaching 11.8 % in 2004 among 15- to 49-year-olds (National Statistical Office (NSO) and ICF Macro, 2004). At the same time, HIV seroprevalence among ANC attendees increased significantly from 1985 to a maximum of 22.8 % in 1999. In 2001, ANC HIV seroprevalence started declining from 16.9 %, reaching 10.6 % in 2010 [10].

## Methodology

### Statistical models

A. Correcting for HIV testing non-response

The first phase of this study is concerned with developing empirical models of individual HIV testing response among those selected to provide blood samples. Since this dependent variable is dichotomous, it is modeled using a probit regression and estimated using maximum likelihood techniques. Suppose there are *I* subnational geographical areas covered by the survey and in each area *n*_*i*_ subjects were selected for HIV testing. Let HIV testing response be defined as *Y*_*ij*_ = 1 if subject *j* in area *i* (*j* = 1,  , *n*_*i*;_; *i* = 1,  *I*) consented to HIV testing and *Y*_*ij*_ = 0 otherwise. The HIV testing response model is defined as

1 $$ {P}_{ij} = Prob\ \left({Y}_{ij}=1\left|{X}_{ij}\right.\right) = \varPhi \left(a + {X}_{ij}\beta \right) $$

where *X*_*ij*_ is a vector of individual and subnational characteristics affecting the individual’s HIV testing response, *α* and *β* are the constant and regression coefficient vectors, respectively; and *Φ* denotes the standard normal cumulative distribution function.

We account for non-response HIV testing by calculating inverse probability weights (IPWs) using the probit model on the pooled data [12]. In order to compute the IPW estimator we estimate probit equations for HIV testing response (*Y*_*ij*_ = 1) versus non-response (*Y*_*ij*_ = 0) from the sample of individuals who were selected to provide blood for HIV testing, conditional on a set of characteristics *X*_*ij*_ that are measured for all HIV testing sampled individuals. This relies on the selection of relevant observables and implies that non-HIV testing response can be treated as ignorable non-response, conditional on *X*_*ij*_ [13]. Selection on the observables requires that *X*_*ij*_ contain variables that predict HIV testing non-response. The covariate variables used in the consent to HIV testing models include individual’s age, gender, educational status, household wealth index, ethnicity, religion, type of place of residence, and region [1, 7, 14, 24]. The probit equation for HIV testing response/non-response is estimated for all HIV selected testing sample, and the inverse of the fitted probabilities from this model, $$ \raisebox{1ex}{$1$}\!\left/ \!\raisebox{-1ex}{${\widehat{P}}_{ij}$}\right. $$ is then used to weight the HIV status data.

B. HIV status prediction model

In the next phase, we developed an HIV prediction model using the observed and known HIV status from the HIV consented and tested subjects. Assume that *m*_*i*_ subjects in subnational area *i* were HIV tested. Let the tested individual’s HIV status be defined as *S*_*ij*_ = 1 if subject *ij* (*j* = 1,  , *m*_*i*;_; *i* = 1,  *I*) tested positive for HIV and *S*_*ij*_ = 0 otherwise. The HIV status prediction model is then defined similarly as

2 $$ {Q}_{ij}=Prob\left({S}_{ij}=1\Big|{Z}_{ij}\right)=\varPhi \left(\delta +{Z}_{ij}\gamma \right) $$

where *Z*_*ij*_ is a vector of individual and subnational characteristics affecting the tested individual’s HIV status, *δ* and *γ* are the constant and regression coefficient vectors, respectively; and *Φ* denotes the standard normal cumulative distribution function. For the subjects who were not tested for HIV, predicted probabilities $\left(\widehat{Q_{ij}}\right)$ of HIV status were estimated using estimated coefficients in (2). Individual-level HIV status (observed or predicted using (2)) are averaged at a desired subnational geographic level to obtain the estimate of HIV prevalence rates. The individual weights used would vary according to use of a) inverse probability weights for those who were selected for HIV testing; b) individual interview weights for those who were interviewed for the main survey but were not selected for the HIV testing sample; and c) household weights for those who were not interviewed and were not in the HIV testing sample [5].

C. A joint spatial model for HIV prevalence

In terms of mapping, the aggregated and weighted population-based HIV prevalence and the antenatal HIV prevalence at the subnational level were used in a shared spatial component. This model was originally developed to map different diseases likely to have similar spatial distributions due to shared risk factors. The model has both shared components and components specific to each disease of interest ([15]; [16, 17]). The shared spatial component model was applied here to incorporate information from both the DHS and ANC HIV data sources. For the ecological model, we took logarithms of the two district-level HIV prevalence rates and adopted an asymmetric formulation of the shared component model – in particular, the asymmetric formulation of the shared spatial component model:

3a $$ \log \left(DHS\_HI{V}_i\right)={\theta}_1+{\beta}_1^T{X}_i+\kappa {u}_i+{v}_{1i} $$

3b $$ \log \left(ANC\_HI{V}_i\right)={\theta}_2+{\beta}_2^T{X}_i+\frac{u_i}{\kappa }+{w}_i+{v}_{2i} $$

where *θ*_*j*_ is the overall HIV risk using DHS data (*j* = 1) and ANC data (*j* = 2), *X*_*j*_ is an ecological covariate risk vector with vector *β*_*j*_ as the corresponding HIV-specific coefficient parameter vector; *u*_*i*_ is the spatially structured shared effect common to both HIV risks measured by DHS and ANC HIV prevalence data; *w*_*i*_ is the ANC structured spatial-specific component, taken as a proxy for excess HIV risk among pregnant women; and *v*_*ji*_ are the HIV source-specific heterogeneous effects, capturing possible extra variation not explained by terms included. Thus, the shared-factor model (3) partitions the risk profile for the two HIV sources into source-specific components and shared spatial components. Parameter *κ* is included to allow for differential gradient on the main shared spatial. The ratio ${\kappa}^{\mathtt{2}}$ compares the risk of DHS HIV source to the risk of ANC source associated with shared spatial component. Ideally, in model (3) we could have split the shared spatial components into unstructured and structured effects or similarly the HIV source-specific components. This seems overly complex and could lead to identifiability problems, so we thought of minimizing the number of random effects. We could have also included specific components for DHS HIV, but such a symmetric formulation can result in identifiability problems.

The statistical smoothing or shrinkage described here could be fitted using frequentist methods, but these are very cumbersome to implement. A number of researchers have advocated Bayesian methods to carry out smoothing of disease risks in spatial epidemiology ([15]; [18]). We modeled both the shared and ANC-specific spatial components using intrinsic conditional (Gaussian) auto-regression (ICAR) models. For instance, *u*_*i*_ is modeled as:

4 $$ f\left({u}_i\left|{u}_k,k\ne i\right.\right)\sim Normal\left(\frac{{\displaystyle {\sum}_{k\in {\varTheta}_i}{W}_{ik}{u}_k}}{{\displaystyle {\sum}_{k\in {\varTheta}_i}{W}_{ik}}},\frac{\lambda_u^2}{{\displaystyle {\sum}_{k\in {\varTheta}_i}{W}_{ik}}}\right) $$

where *Θ*_*i*_ is the set of areas adjacent to area *i*; *W*_*ik*_ is the weight reflecting spatial dependence between areas *i* and *k* and *λ*_*u*_^2^ is the (conditional) structured variance. The most common and simplest adjacent specification is to set *W*_*ik*_ = 1 if areas *i* and *k* are neighbors that share a common boundary and *W*_*ik*_ = 0 otherwise. Thus, a CAR normal prior specifies the conditional distribution of each area-specific effect, given all the neighboring effects, to be a normal distribution with mean equal to the average of spatial effect of its neighbors and variance inversely proportional to the number of neighbors; the more neighbors an area has, the greater the precision for that area effect. This might also be reflected for population density, where urban areas may have more neighbors than sparsely populated rural areas. Since we are using the CAR normal prior, with sum-to-zero constraints on the random effect terms, we assign flat normal priors on the overall intercept and fixed-effect terms. The logarithm of the scaling parameters log *k* is assigned a $\mathtt{Normal}\;\left(\mathtt{0},\kern0.5em \mathtt{5}\right)$ prior distribution. All precision parameters are assigned independent hyper-prior $\mathtt{Gamma}\;\left(\mathtt{0.5},\kern0.5em \mathtt{0.0005}\right)$ distributions. These prior specifications have been shown in spatial modeling literature to provide plausible range for relative risk assumptions (see, for example, Manda et al. [19]). Model evaluation and validation were done using the conditional predictive ordinate (CPO) [20], which is the marginal posterior predictive density. This is also known as leave-one-out validation, where a large CPO indicates agreement between observations and the model.

The Bayesian estimation of the model parameters was carried out by running three parallel Gibbs sampler chains for 20,000 iterations from independent starting positions. Using a combination of trace plots and formal convergence diagnosis tools, satisfactory convergence was achieved by 5,000 iterations in each case. Posterior summaries were based on a combined sample of the remaining 45,000 iterations needed to complete the cycles. An abridged copy of the WinBUGS code used for fitting the bivariate spatial model is provided in the Appendix.

### Data sources

The first data that were analyzed for this study were from the Malawi Demographic and Health Survey (MDHS) of 2010 (MDHS 2010). This was a nationally representative household survey that provided data for a wide range of population, health, and nutrition indicators. Malawi is one of only a few countries where a Demographic and Health Survey has collected nationally representative HIV prevalence data (NSO, Malawi and ICF Macro, 2011). The survey sampled a total of 27,000 households and involved nearly 24,000 female and 7,000 male respondents. In every third household, blood specimens were collected for HIV testing from all women aged 15–49 and men aged 15–54 who consented to HIV testing. Both the weighting and the prediction model as described above in the statistical models are based on this dataset.

The second dataset, from a study conducted in Malawi in 2010, concerned HIV prevalence among pregnant women in Malawi. ANC HIV surveillance in Malawi has been conducted every one to two years since 1994 using a consistent methodology in the same population group. For the 2010 ANC survey, a total of 23,788 pregnant women were enrolled from 28 urban sites and 26 rural sites distributed across the three regions of Malawi. This was in line with the decentralization process, and as such sufficient numbers were needed for HIV data to assist in developing district-specific plans [21].

The mapping modeling was conducted at the district level, the level of geographic aggregation at which primary health care is conducted. There are currently a total of 28 administrative districts in Malawi. However, due to two districts being so small, we combined them into the original districts from which they separated around 2000. Two contextual variables were modeled at the district level: i) the level of poverty [22], defined as the total annual per capita consumption reported by a household, and ii) the population density [23], defined as number of people per square kilometer (used as a proxy for social mobility and interactions, which fuels increased HIV and STI transmission rates) [19]. Each of these contextual covariates was partitioned into fourths; this categorization enables effects to be detected at the extremes of the range.

## Results

**Fig. 1:**
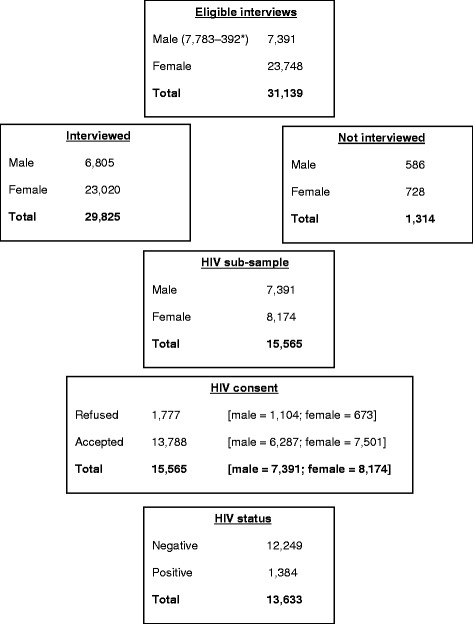
Total sample categories: males and females 15–49 years old *392 men were aged 50–54 and were consequently removed from this analysis for comparative analyses between women and men in DHS and ANC

The results for the response rates for households, individuals, and HIV testing in the MDHS 2010 are shown diagrammatically in Fig. 1. A total of 27,307 households were selected, and of these, 24,825 were successfully interviewed, yielding a response rate of 98 %. Of the eligible 23,748 and 7,391 women and men (aged 15–49 years), 23,020 and 6,805 were interviewed, respectively. This resulted in 97 and 92 % response rates for women and men, respectively. In the 1:3 households selected for HIV testing, 1,104 of the 7,391 selected men and 673 of the 8,174 selected women did not consent to HIV testing, resulting in an overall 89 % consent rate for HIV testing. However, this reduces to only 52 % of all interviewed adults.

**Fig. 2:**
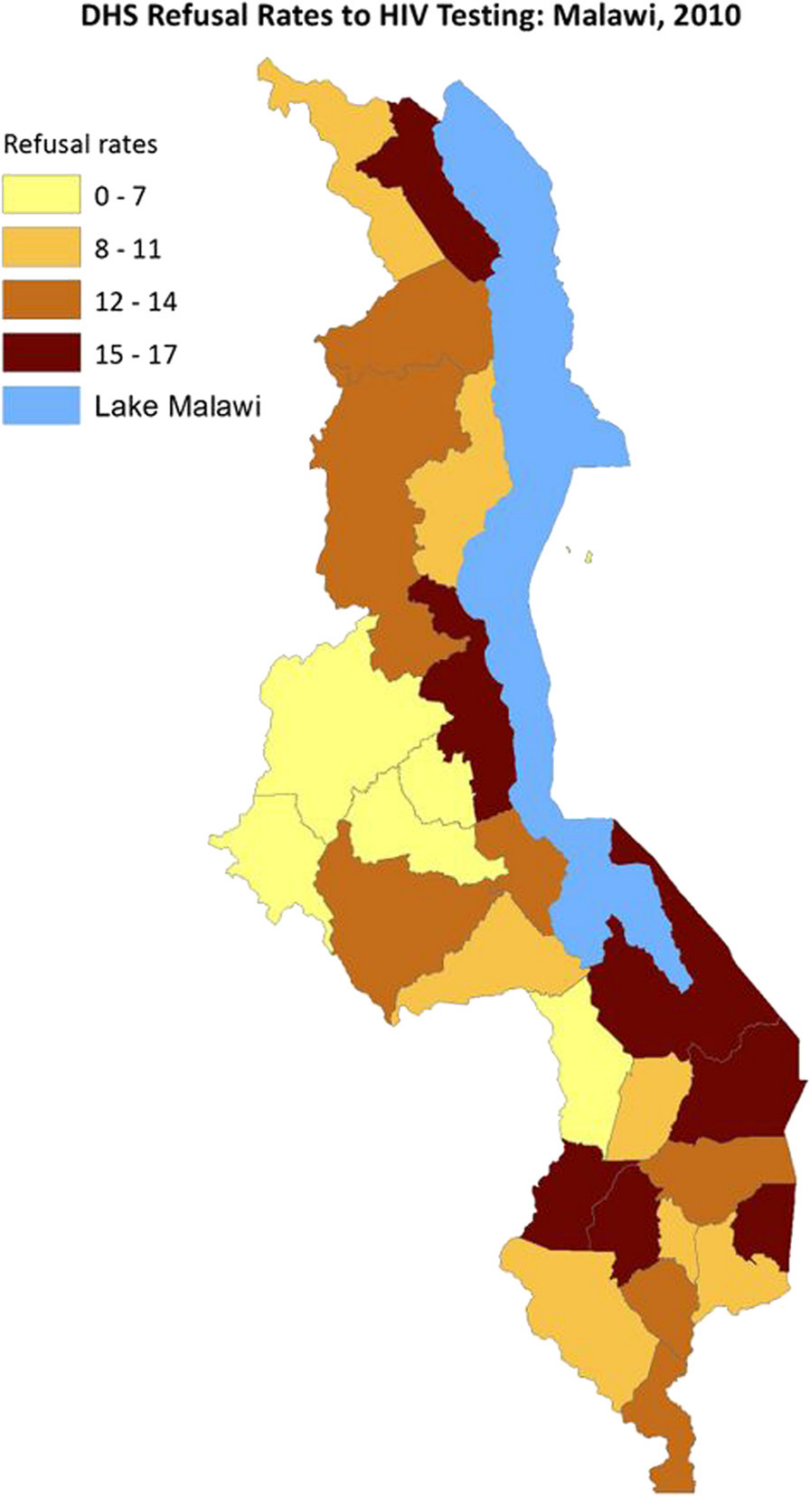
DHS Refusal Rates to HIV Testing MAlawi, 2010

Table 1 presents the coverage rates for HIV testing and the estimated coefficients from fitting the probit regression model to HIV testing response by sex, urban–rural residence, region, and many other socioeconomic factors. Females (92 %) rather than males (85 %) and rural (89 %) rather than urban (85 %) residence are significantly associated with higher rates of consenting to HIV testing. Being employed in agriculture, skilled manual labor, and other kinds of work have a significant positive association with acceptance of HIV testing. Other Christians and Muslims are significantly less likely to accept HIV testing. Though increased level of education leads to more acceptance of HIV testing, it was not significant. Other marital status categories were negatively associated with HIV testing compared to being single, but again not significantly. No clear pattern emerged for age and household wealth. The distribution of HIV testing refusal rates is shown in Fig. 2, where higher refusal rates of HIV testing were observed in the southeastern and in most northern parts of the country. The adjusted coefficients in Table 1 were used to derive the inverse probability weighting as described earlier. The final weights for all sampled subjects had a mean of 1.04 (IQR range 0.78-1.10), showing very few outlier weights.

**Table 1:** Distribution of subjects by HIV testing response, with corresponding coefficient estimates and 95 % confidence intervals from the probit regression model among subjects selected for HIV testing, MDHS 2010

	Sub-sample selected for HIV testing (%)		Regression coefficients (95 % CI)	
	Tested	Total	Unadjusted	Adjusted
Overall	13,788 (88.58)	15,565 (100)		1.23 (1.01; 1.45)
Gender				
Male	6,287 (85.06)	7,391 (47.48)	0.0	0.0
Female	7,501 (91.77)	8,174 (52.52)	0.35 (0.30; 0.40)	0.25 (0.17; 0.32)
Age (years)				
15–19	3,319 (87.73)	3,783 (24.30)	0.0	0.0
20–24	2,569 (88.65)	2,898 (18.62)	0.05 (−0.03; 0.13)	−0.04 (−0.15; 0.08)
25–29	2,396 (89.07)	2,690 (17.28)	0.07 (−0.01; 0.15)	−0.06 (−0.19; 0.08)
30–34	1,882 (87.25)	2,157 (13.86)	−0.02 (−0.11; 0.06)	−0.13 (−0.27; 0.01)
35–39	1,544 (89.40)	1,727 (11.10)	0.09 (−0.01; 0.18)	0.01 (−0.14; 0.16)
40–44	1,093 (89.96)	1,215 (7.81)	0.12 (0.01; 0.23)	0.01 (−0.15; 0.18)
45–49	985 (89.95)	1,095 (7.04)	0.12 (0.004; 0.231)	−0.05 (−0.22; 0.12)
Residence				
Urban	1,885 (84.83)	2,222 (14.28)	0.0	0.0
Rural	11,903 (89.21)	13,343 (85.72)	0.21 (0.14; 0.28)	0.16 (0.06; 0.26)
Education				
No education	1,485 (87.71)	1,693 (10.88)	0.0	0.0
Primary	9,097 (89.21)	10,197 (65.51)	0.08 (−0.01; 0.16)	0.05 (−0.06; 0.16)
Secondary plus	3,172 (87.48)	3,626 (23.30)	−0.01 (−0.10; 0.08)	0.07 (−0.06; 0.21)
Missing	34 (69.39)	49 (0.31)	−0.65 (−1.03; −0.28)	0.04 (−0.58; 0.66)
Employment				
Not working	2,780 (92.67)	3,000 (20.39)	0.0	0.0
Professional/tech/manag	297 (90.83)	327 (2.22)	−0.12 (−0.32; 0.08)	−0.01 (−0.23; 0.21)
Sales	1,708 (91.88)	1,859 (12.63)	−0.05 (−0.16; 0.05)	−0.01 (−0.12; 0.10)
Agriculture	6,244 (93.88)	6,651 (45.20)	0.09 (0.01; 0.18)	0.11 (0.02; 0.20)
Skilled manual	1,249 (92.93)	1,344 (9.13)	0.02 (−0.10; 0.14)	0.14 (0.01; 0.27)
Others	1,433 (93.36)	1,535 (10.43)	0.05 (−0.07; 0.17)	0.14 (0.01; 0.26)
Marital status				
Married	8,530 (89.72)	9,507 (61.08)	0.0	0.0
Divorced	865 (90.39)	957 (6.15)	0.04 (−0.08; 0.15)	−0.04 (−0.17; 0.10)
Widowed	306 (91.07)	336 (2.16)	0.08 (−0.11; 0.27)	−0.10 (−031; 0.12)
Single	3,965 (85.79)	4,622 (29.69)	−0.20 (−0.25; −0.14)	−0.04 (−0.15; 0.07)
Missing	122 (85.31)	143 (0.92)	−0.22 (−0.47; 0.04)	0.13 (−0.23; 0.50)
Wealth				
Poorest	2,391 (87.78)	2,724 (17.50)	0.0	0.0
Poorer	2,805 (89.47)	3,135 (20.14)	0.09 (0.004; 0.173)	0.08 (−0.02; 0.19)
Middle	2,838 (89.95)	3,155 (20.27)	0.12 (0.03; 0.20)	0.06 (−0.04; 0.16)
Richer	2,907 (88.95)	3,268 (21.00)	0.06 (−0.02; 0.14)	0.07 (−0.03; 0.18)
Richest	2,847 (86.72)	3,283 (21.09)	−0.05 (−0.13; 0.03)	−0.003 (−0.12; 0.12)
Religion				
Catholic	2,942 (94.51)	3,113 (21.15)	0.0	0.0
CCAP	2,218 (94.58)	2,345 (15.94)	0.01 (−0.10; 0.12)	0.03 (−0.08; 0.14)
Anglican	440 (93.62)	470 (3.19)	−0.08 (−0.27; 0.12)	−0.06 (−0.26; 0.13)
Seventh Day	1,025 (93.95)	1,091 (7.41)	−0.05 (−0.19; 0.09)	−0.03 (−0.17; 0.11)
Other Christian	5,411 (93.05)	5,815 (39.51)	−0.12 (−0.21; −0.03)	−0.13 (−0.22; −0.05)
Muslim	1,372 (88.01)	1,559 (10.59)	−0.42 (−0.53; −0.32)	−0.42 (−0.53; −0.30)
No religion	206 (93.21)	221 (1.50)	−0.11 (−0.37; 0.16)	−0.05 (−0.32; 0.22)
Missing	97 (95.10)	102 (0.69)	0.06 (−0.36; 0.47)	0.09 (−0.34; 0.52)

The observed unweighted MDHS 2010 HIV prevalence was 10.15 %, with a 95 % confidence interval (CI) of 9.66 to 10.67 %, while the weighted prevalence (using MDHS 2010 HIV response weight) was 10.61 % (9.9, 11.33 %). Using the inverse probability weighting (IPW), the MDHS 2010 HIV prevalence was 10.19 % (9.69 %, 10.71 %). On the other hand, ANC 2010 IV prevalence per site had a median of 10.6 % (1.85 to 24.09 %). The district was chosen as an appropriate level to depict the geographical distribution of the HIV prevalence. The distribution of the MDHS 2010 sample (ages 15–49) and ANC 2010 attendees per district ranged from 413–877 and from 297 to 1,762, respectively. The specific data source district-level HIV prevalence is shown in Table 2. In most cases the specific HIV prevalence confidence intervals overlap, meaning that the estimates are not statistically different. The district-level HIV prevalence for MDHS 2010 had mean and median of 10.45 and 10.09 % and ranged from 3.16–18.00 %; for ANC 2010, district-level HIV prevalence had a mean and median of 12.12 and 11.01 % and ranged from 5.54–25.2 %. This showed great variation in HIV prevalence rates between the districts, where some estimates were based on very small sample sizes.

**Table 2:** District-level HIV prevalence from antenatal clinics (ANCs) and nationally representative surveys, Malawi 2010

District	ANC HIV	DHS HIV
Balaka	11.58 (10.45 – 12.70)	14.14 (11.09 – 17.85)
Blantyre	17.49 (16.48 – 18.50)	15.96 (13.12 – 19.27)
Chiradzulu	20.26 (18.88 – 21.65)	16.47 (13.30 – 20.21)
Dowa	6.08 (5.26 – 6.90)	3.76 (2.29 – 6.13)
Nkhata Bay	11.14 (10.04 – 12.23)	10.18 (7.69 – 13.35)
Nkhotakota	7.30 (6.14 – 8.46)	5.21 (0.35 – 0.78)
Ntchisi	5.54 (4.74 – 6.34)	3.66 (2.21 – 6.01)
Salima	8.61 (7.63 – 9.59)	8.85 (6.06 – 12.75)
Rumphi	9.90 (8.88 – 10.93)	6.67 (0.47 – 9.46)
Mzimba	9.10 (8.38 – 9.82)	5.08 (3.26 – 7.84)
Kasungu	8.50 (7.34 - 9.66)	4.62 (0.31 - 6.80)
Mchinji	10.13 (9.09 – 11.17)	0.96 (7.09 – 12.96)
Lilongwe	11.90 (11.13 – 12.68)	9.35 (6.92 – 12.53)
Dedza	9.80 (8.60 – 11.00)	0.75 (5.33 – 10.51)
Ntcheu	8.80 (7.16 – 10.44)	12.16 (9.46 – 15.51)
Chikwawa	11.42 (10.15 – 12.70)	10.96 (7.70 – 15.37)
Nsanje	16.30 (14.65 – 17.95)	15.06 (11.71 – 19.15)
Thyolo	25.20 (23.33 – 27.07)	18.00 (14.42 – 22.22)
Mulanje	19.50 (18.13 – 20.88)	17.11 (13.89 – 20.89)
Phalombe	15.87 (14.78 – 16.96)	1.46 (11.67 – 18.08)
Machinga	14.27 (12.94 – 15.60)	14.34 (10.69 – 18.97)
Mangochi	10.89 (9.79 – 11.99)	10.01 (7.32 – 13.53)
Zomba	15.50 (14.35 – 16.66)	14.98 (11.84 – 18.77)
Karonga	11.50 (10.40 – 12.59)	9.82 (7.20 – 13.25)
Chitipa	7.97 (7.24 – 8.70)	3.17 (1.74 – 5.69.)
Mwanza/Neno	10.43 (9.49 – 11.38)	1.05 (8.11 – 13.36)

The observed MDHS 2010 HIV prevalence by various characteristics of subjects who consented to HIV testing is shown in Table 3, together with the estimated probit regression coefficients for the HIV status prediction model. A number of factors, such as gender, residence, employment, marital status, ethnicity, condom use, multiple sex partners, and risky sex in the past 12 months, are significantly associated with HIV status. The discriminative ability of the resultant model had area under the receiver operating curve (AROC) of 0.7757, 95 % CI (0.7680, 0.7833 %), which is deemed satisfactory. After predicting HIV status for the non-tested (31,139 - 13,633 = 17,406) subjects, and assigning to all the subjects their differing weights as described above, the overall HIV prevalence using all the 31,139 subjects was estimated at 11.05 % (10.80, 11.30 %).

**Table 3:** HIV prevalence, with corresponding coefficient estimates and 95 % confidence intervals from probit regression model among subjects who accepted HIV testing, MDHS 2010

	Frequencies for sub-sample with HIV results (%)			Regression coefficients (95 % CI)	
	Negative – n (%)	Positive – n (%)	Total	Unadjusted	Adjusted
Overall	12,249 (89.84)	1,384 (10.16)	13,633		−2.05 (−2.31; −1.80)
Gender					
Male	5,716 (92.06)	493 (7.94)	6,208	0.0	0.0
Female	6,533 (87.99)	892 (12.01)	7,425	0.24 (0.18; 0.29)	0.22 (0.14; 0.30)
Age (years)					
15–19	3,217 (97.81)	72 (2.19)	3,289	0.0	0.0
20–24	2,413 (95.00)	127 (5.00)	2,540	0.37 (0.25; 0.50)	0.21 (0.04; 0.38)
25–29	2,131 (90.18)	232 (9.82)	2,363	0.72 (0.61; 0.84)	0.50 (0.33; 0.67)
30–34	1,575 (84.63)	286 (15.37)	1,861	1.00 (0.88; 1.11)	0.77 (0.60; 0.94)
35–39	1,223 (80.41)	298 (19.59)	1,521	1.16 (1.04; 1.28)	0.96 (0.78; 1.13)
40–44	876 (80.66)	210 (19.34)	1,086	1.15 (1.02; 1.28)	0.91 (0.72; 1.09)
45–49	814 (83.66)	159 (16.34)	973	1.04 (0.90; 1.17)	0.81 (0.62; 1.00)
Residence					
Urban	1,545 (82.53)	327 (17.47)	1,872	0.0	0.0
Rural	10,704 (91.00)	1,058 (9.00)	11,761	−0.41 (−0.48; −0.33)	−0.41 (−0.50; −0.32)
Education					
No education	1,270 (86.81)	193 (13.19)	1,463	0.0	0.0
Primary	8,141 (90.46)	859 (9.54)	9,000	−0.19 (−0.28; −0.10)	0.07 (−0.03; 0.17)
Secondary plus	2,810 (89.58)	327 (10.42)	3,137	−0.14 (−0.24; −0.04)	0.11 (−0.02; 0.24)
Missing	28 (84.85)	5 (15.15)	33	0.09 (−0.44; 0.61)	0.23 (−0.37; 0.82)
Employment					
Not working	2,571 (93.46)	180 (6.54)	2,751	0.0	0.0
Professional/tech/manag	236 (80.00)	59 (20.00)	295	0.67 (0.49; 0.85)	0.23 (0.02; 0.44)
Sales	1,426 (84.53)	261 (15.47)	1,687	0.49 (0.39; 0.60)	0.20 (0.07; 0.32)
Agriculture	5,672 (91.81)	506 (8.19)	6,178	0.12 (0.03; 0.20)	−0.01 (−0.11; 0.10)
Skilled manual	1,070 (86.22)	171 (13.78)	1,241	0.42 (0.31; 0.53)	0.21 (0.07; 0.35)
Others	1,215 (85.62)	204 (14.38)	1,419	0.45 (0.34; 0.56)	0.20 (0.07; 0.33)
Marital status					
Married	7,505 (89.01)	927 (10.99)	8,432	0.0	0.0
Divorced	648 (75.97)	205 (24.03)	853	0.52 (0.42; 0.62)	0.39 (0.25; 0.52)
Widowed	158 (52.15)	145 (47.85)	303	1.17 (1.03; 1.32)	0.83 (0.64; 1.01)
Single	3,824 (97.43)	101 (2.57)	3,925	−0.72 (−0.81; −0.63)	−0.37 (−0.54; −0.19)
Missing	114 (95.00)	6 (5.00)	120	−0.42 (−0.80; −004)	−0.09 (−0.59; 0.40)
Religion					
Catholic	2,625 (90.39)	279 (9.61)	2,904	0.0	0.0
CCAP	2,018 (91.56)	186 (8.44)	2,204	−0.07 (−0.17; 0.03)	−0.02 (−0.13; 0.10)
Anglican	385 (88.91)	48 (11.09)	433	0.08 (−0.09; 0.25)	0.14 (−0.06; 0.34)
Seventh Day	885 (87.71)	124 (12.29)	1,009	0.14 (0.03; 0.26)	0.06 (−0.08; 0.19)
Other Christian	4,796 (89.48)	564 (10.52)	5,360	0.05 (−0.03; 0.13)	0.05 (−0.04; 0.14)
Muslim	1,212 (88.66)	155 (11.34)	1,367	0.10 (−0.01; 0.20)	−0.05 (−0.21; 0.12)
No religion	184 (92.00)	16 (8.00)	200	−0.10 (−0.36; 0.16)	−0.21 (−0.52; 0.10)
Missing	85 (90.43)	9 (9.57)	94	−0.002 (−0.36; 0.35)	0.18 (−0.19; 0.56)
Ethnicity					
Chewa	3,812 (93.55)	263 (6.45)	4,075	0.0	0.0
Tumbuka	1,269 (93.04)	95 (6.96)	1,364	0.04 (−0.08; 0.16)	−0.07 (−0.20; 0.07)
Lomwe	1,921 (83.67)	375 (16.33)	2,296	0.54 (0.45; 0.62)	0.54 (0.44; 0.64)
Tonga	431 (89.98)	48 (10.02)	479	0.24 (0.07; 0.40)	0.16 (−0.03; 0.35)
Yao	1,193 (87.27)	174 (12.73)	1,367	0.38 (0.27; 0.48)	0.38 (0.23; 0.54)
Sena	673 (88.20)	90 (11.80)	763	0.33 (0.20; 0.46)	0.34 (0.19; 0.49)
Nkhonde	199 (91.71)	18 (8.29)	217	0.13 (−0.12; 0.38)	−0.08 (−0.36; 0.21)
Ngoni	1,659 (89.24)	200 (10.76)	1,859	0.28 (0.18; 0.38)	0.21 (0.10; 0.32)
Mang’anja	311 (85.44)	53 (14.56)	364	0.46 (0.29; 0.63)	0.43 (0.24; 0.61)
Others	346 (88.72)	44 (11.28)	390	0.31 (0.13; 0.48)	0.21 (0.02; 0.41)
Missing	376 (94.71)	21 (5.29)	397	−0.10 (−0.31; 0.11)	−0.23 (−0.47; 0.01)
Condom use					
No	7,712 (90.20)	838 (9.80)	8,550	0.0	0.0
Yes	1,191 (82.02)	261 (17.98)	1,452	0.38 (0.29; 0.46)	0.59 (0.49; 0.69)
Missing	1,404 (84.63)	255 (15.37)	1,659	0.27 (0.19; 0.35)	0.17 (0.02; 0.32)
Age at first sex					
Less than 15	2,037 (88.14)	274 (11.86)	2,311	0.0	0.0
15–19	6,082 (88.43)	796 (11.57)	6,878	−0.01 (−0.09; 0.06)	−0.07 (−0.16; 0.01)
20 and above	1,754 (89.90)	197 (10.10)	1,951	−0.09 (−0.19; 0.01)	−0.21 (−0.33; 0.10)
Missing	434 (83.30)	87 (16.70)	521	0.22 (0.07; 0.36)	−0.01 (−0.17; 0.15)
Multiple sex partners					
No	8,198 (89.08)	1,005 (10.92)	9,203	0.0	0.0
Yes	573 (86.17)	92 (13.83)	665	0.14 (0.02; 0.27)	0.27 (0.08; 0.47)
No sex past 12 months	1,536 (85.71)	256 (14.29)	1,792	0.16 (0.08; 0.24)	0.01 (−0.12; 0.14)
Risk sex in 12 months					
No incl. no sex past 12 months	8,562 (88.20)	1,146 (11.80)	9,708	0.0	0.0
Yes	1,730 (89.31)	207 (10.69)	1,937	−0.06 (−0.14; 0.02)	−0.05 (−0.22; 0.12)

**Table 4:** Estimated covariate effects with associated 95 % credible intervals using the Bivariate Spatial Analysis: Malawi 2010

Characteristics	DHS HIV	ANC HIV
Poverty fourths		
I (Lowest)	0.0	0.0
II	0.11 (−0.18, 0.40)	0.16 (−0.08, 0.39)
III	0.10 (−0.18, 0.36)	0.24 (0.01, 0.45)
IV (Highest)	−0.00026 ( −0.31, 0.31)	0.17 (−0.09, 0.42)
Population density fourths		
I (Lowest)	0.0	0.0
II	−0.09 (−0.41, 0.26)	−0.11 (−0.38, 0.16)
III	0.09 (−0.19, 0.38)	−0.06 (−0.29, 0.18)
IV (Highest)	0.34 (0.02, 0.65)	0.37 (0.11, 0.63)

**Fig. 3:**
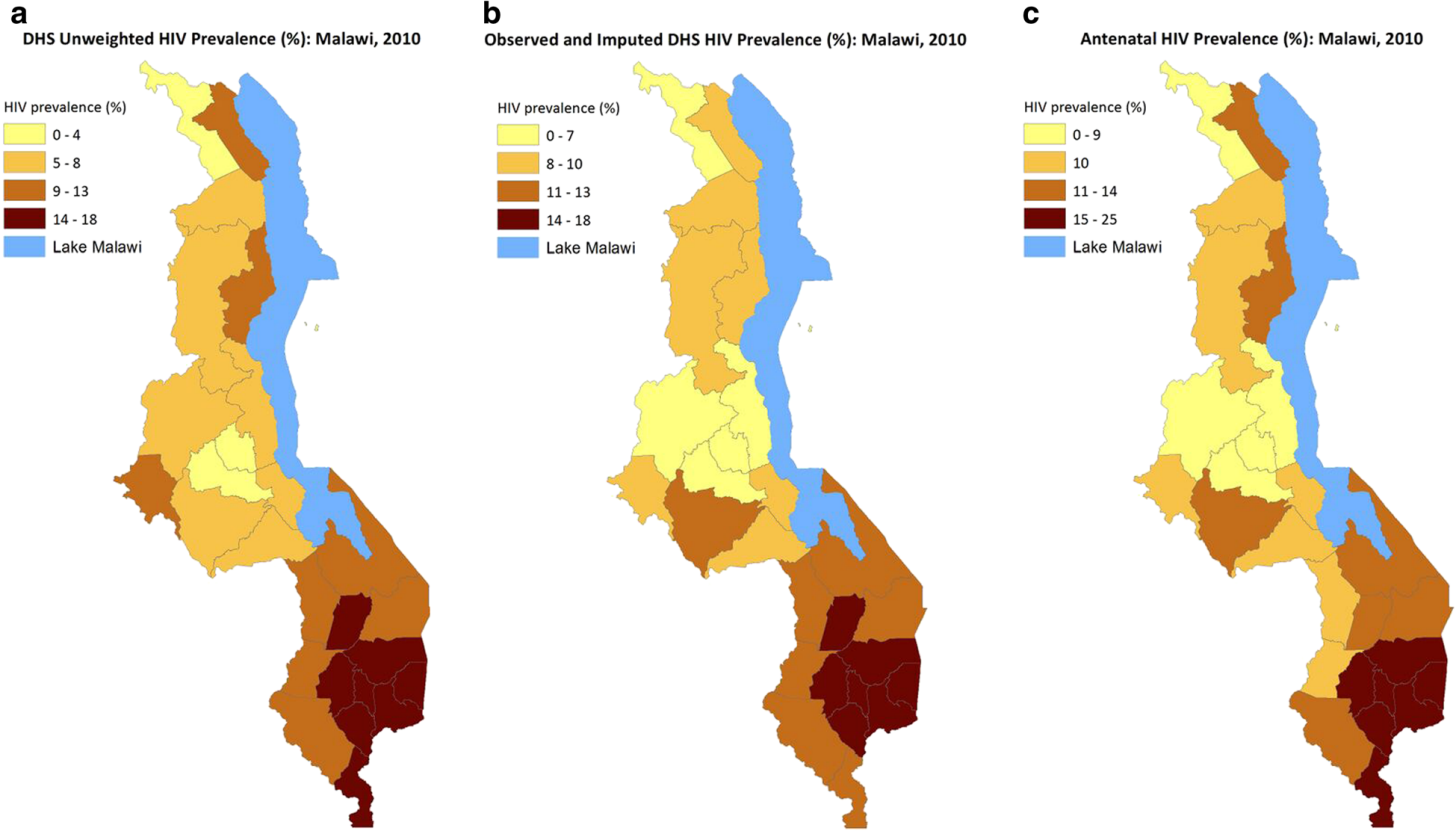
**a** DHS unweighted HIV Prevalence (%): Malawi, 2010. **b** Observed + Imputed DHS HIV Prevalence (%): MAlawi, 2010. **c** Antenatal HIV Prevalence (%): Malawi, 2010

**Fig. 4:**
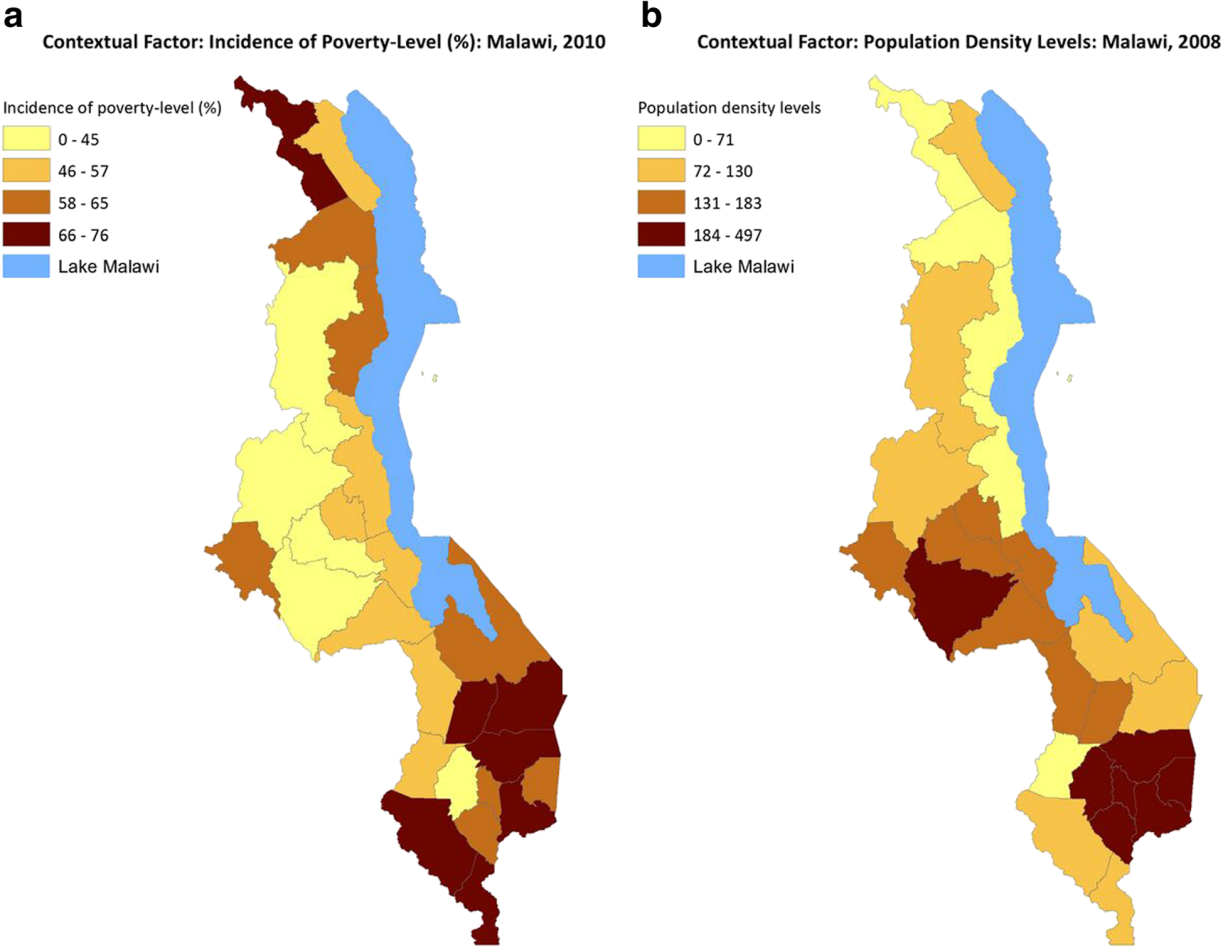
**a** Contextual Factor: Incidence of Poverty-Level (%): Malawi,2011. **b** Contextual Factor Population Density Levels: Malawi, 2008

Maps in Fig. 3a-b show DHS unweighted and total weighted (all 31,139 subjects) HIV prevalence, and the antenatal HIV prevalence in Malawi 2010. There appear to be more districts, especially in the central western district, where after adjustments the HIV rates have increased. The distributions of the two contextual factors are shown in Fig. 4a-b. Higher MDHS 2010 HIV prevalence rates were observed in the mostly southern districts, which mirrors the ANC HIV that is mostly concentrated in the southeastern districts. Poverty level is evenly spread, but southern districts bear the most burden of poverty. The same goes for population density. Thus districts with high HIV prevalence have high levels of poverty and population density.

We fitted univariate spatial models controlling for the two contextual factors and both the structured and unstructured district-level random spatial effects. However, a bivariate spatial model using the shared-component technique was more adequate than separate spatial DHS and ANC HIV prevalence models. The results of the effects of the contextual factors from fitting the smoothed risks using the bivariate shared model are shown in Table 4. High population density areas were significantly associated with high HIV prevalence. Increased district-level poverty was associated with increased HIV rates, but this was not statistically significant.

**Fig. 5:**
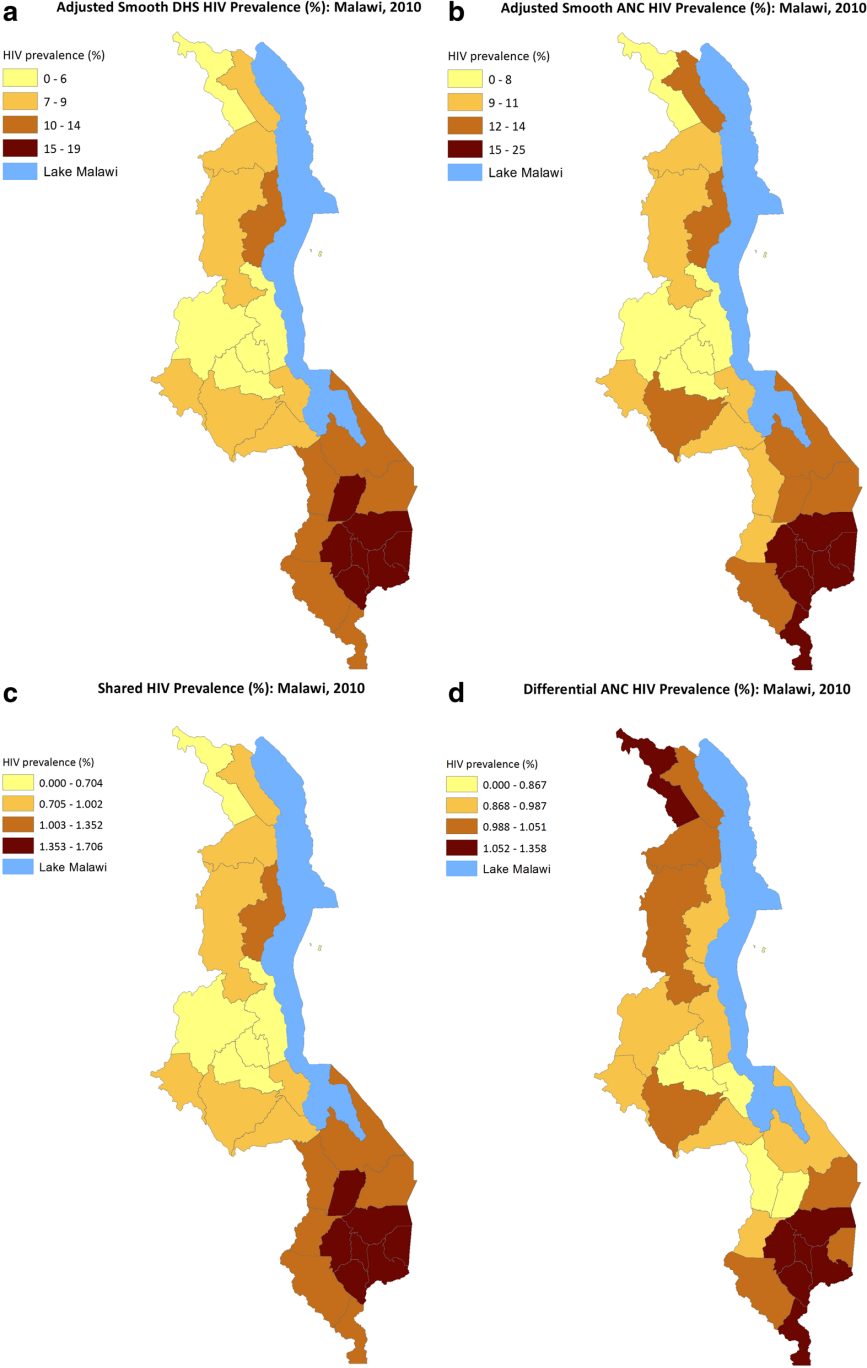
**a** Adjusted Smooth DHS HIV Prevalence (%): Malawi, 2010. **b** Adjusted Smooth ANC HIV Prevalence (%): MAlawi, 2010. **c** Shared HIV Prevalence (%): Malawi, 2010. **d** Differential ANC HIV Prevalence (%): Malawi, 2010

The covariate-adjusted smoothed maps of HIV risk from fitting the bivariate spatial model are shown in Fig. 5a-d. The estimated HIV-specific components in Fig. 5a and b are generally similar to the observed and weighted prevalence maps (Fig. 3a-c), and clusters of high HIV-prevalent districts are now more apparent. The estimates of the effects of the shared component (which we took to act as a surrogate for high HIV risky behaviors) had a larger effect on HIV incidence in the southern parts of the country around the high population density and urban areas. The excess risk attributable to ANC HIV was much larger in the central-eastern and northern parts of the country.

## Conclusions

In this paper, we have proposed a novel approach to estimating HIV prevalence using a nationally representative population-based survey and antenatal clinic sentinel survey. The methodology has used a suite of techniques aimed at overcoming some non-HIV testing response biases in national surveys and the coverage limitations inherent in antenatal surveys. In particular, our approach could prove very useful where there is need to produce subnational HIV prevalence estimates using national probability and representative samples, which might not have adequate numbers of tested individuals due to refusals to consent to HIV testing. Even though a nationally representative population-based survey remains the best source of HIV data [4], its usefulness can be enhanced by correctly accounting for non-response to HIV testing and by jointly modeling antenatal clinics’ HIV data, often the only source of HIV prevalence. This has enriched the amount of HIV data available to increase power and precision of the estimates.

We have found that adjusting the observed national HIV prevalence rates to account for those non-tested individuals resulted in rates slightly higher than the observed prevalence. The adjusted national HIV prevalence was estimated at 11.05 % (10.80, 11.30 %), while the other estimates based on tested individuals were 10.61 % (9.9, 11.33 %) and 10.19 % (9.69, 10.71 %) using the survey HIV weight and the inverse probability weighting (IPW), respectively; the latter estimate was significantly lower. These results are partially consistent with analyses of effects of non-testing response to HIV prevalence estimates using similar DHS surveys with HIV testing; these showed non-significant effect of non-HIV testing on national HIV prevalence estimates [5, 6]. Our results show a slight significant increase, confirming the hypothesis that HIV testing refusals may have higher HIV prevalence. However, these results should be treated with caution as they may depend on the type of adjustment and prediction models and the set of socio-demographic and behavioral characteristics included in the models. At AROC of 0.78, our HIV prediction was thought to be very satisfactory using in-sample validation.

A few studies have appeared in the literature looking at assessing and accounting non-response in estimating HIV prevalence in Malawi. Obare [24] and Floyd et al. (2013) analyzed repeated HIV serosurveys in rural Malawi and found that HIV testing refusal rates were at 45 and 43 % for men and women in subsequent surveys, respectively. As outlined in Obare [24], a downward bias in the HIV prevalence estimates can result from many factors, including prior knowledge of HIV-positive status, migration, spouses’/partners’ infidelity suspicion, and worries about getting HIV/AIDS. In the absence of true HIV estimate among non-tested subjects, these conjectures are just indicative of the down effect of nonresponse on the HIV prevalence estimate. In some cases, researchers including Obare [24] have investigated the impact of non-response on HIV prevalence estimates by assuming plausible prevalence rates among non-participants where a significant downward bias was shown in HIV estimates among individuals who were tested in all repeated surveys. In the present study, however, we explicitly predicted HIV status for those not tested, and the prediction model was deemed very satisfactory.

A recent study by Zulu et al. [10] used Geographic Information Systems (GIS) software to map HIV prevalence obtained from ANC surveys year by year from 1995. Additionally, they used spatial autocorrelation, clustering measures, and multiple regression analyses of the 2010 ANC data. Five socio-demographic, behavioral, sociobiological, and geographic variables were found to be significantly associated with HIV prevalence in multiple ordinary least squares (OLS) regression analysis. Their spatial variation at the district level was mapped in relation to the spatial distribution of HIV hotspots, cold spots, and spatial outliers.

A study that proposes a methodology with similarities to what has been presented here can be found in Ivaschenko and Lanjouw [2]. They also combined HIV data from sentinel surveillance survey of 2001 with representative population-based HIV data from 2000. While we model both in joint mapping models, they use the latter to weight the ANC data, which is used to generate an HIV prediction model. This model is then used to predict HIV status in MDHS 2000 sampled individuals. It’s critical to note that the MDHS 2000 did not include HIV testing at all. The predicted HIV prevalence for the women in the MDHS 2000 was then aggregated to the district-level prevalence and compared to the observed HIV prevalence at the same level obtained in MDHS 2004 as a check of the validity and accuracy of the prediction HIV model. Our approach improved on these three by combining spatial smoothing techniques based on novel application of multivariate spatial models, inverse probability weighting, and HIV prediction model.

In conclusion, minimizing nonresponse is a major challenge for all population-based surveys. As argued in [24] and Floyd et al. [1], in populations where most know their HIV status, population-based prevalence estimates can be heavily biased. In such situations, high-coverage antenatal clinics’ surveillance HIV data are the only main sources. However, we have shown that inverse probability weighting can be used to correct for non-response to HIV testing, especially if the number of tested individuals is very minimal at the subnational level.

## Appendix

### WinBUGS Code

The WinBUGS code for the main model, involving the bivariate district MDHS 2010 and ANC 2010 HIV prevalence rates, is provided here for reference. The nodes adj, weights and num are a vector listing the ID numbers of the adjacent areas for each district; a vector of the same length as adj that provides unnormalized weights associated with each pair of districts, and a vector giving the number of neighbors for each district. All the three can be generated using the Adjacency Tool in GeoBUGS, an add-on mapping tool to WinBUGS.
